# Catastrophic outcomes of thrombus ‘bumper’ ball in neglected severe rheumatic mitral stenosis: report of three cases

**DOI:** 10.1093/jscr/rjad266

**Published:** 2023-05-18

**Authors:** Zineb Agoumy, Houda Bachri, Samah E L Mhadi, Hamza Chraibi, Zineb F Fehri, Lamyaa Bakamel, Hala El Assili, Kaoutar Berrag, Sanae Es-sebany, Hasna Leghlimi, Fadwa Lachhab, Mohamed Tribak, Adil Bensouda, Ali Chaib, Nesma Bendagha, Aida Soufiani, Said Moughil

**Affiliations:** Cardiovascular Department B, Ibn Sina Hospital, Mohammed V University, Rabat, Morocco; Cardiovascular Department B, Ibn Sina Hospital, Mohammed V University, Rabat, Morocco; Cardiovascular Department B, Ibn Sina Hospital, Mohammed V University, Rabat, Morocco; Cardiovascular Department B, Ibn Sina Hospital, Mohammed V University, Rabat, Morocco; Cardiovascular Department B, Ibn Sina Hospital, Mohammed V University, Rabat, Morocco; Cardiovascular Department B, Ibn Sina Hospital, Mohammed V University, Rabat, Morocco; Cardiovascular Department B, Ibn Sina Hospital, Mohammed V University, Rabat, Morocco; Cardiovascular Department B, Ibn Sina Hospital, Mohammed V University, Rabat, Morocco; Cardiovascular Department B, Ibn Sina Hospital, Mohammed V University, Rabat, Morocco; Cardiovascular Department B, Ibn Sina Hospital, Mohammed V University, Rabat, Morocco; Cardiovascular Department B, Ibn Sina Hospital, Mohammed V University, Rabat, Morocco; Cardiovascular Department B, Ibn Sina Hospital, Mohammed V University, Rabat, Morocco; Cardiovascular Department B, Ibn Sina Hospital, Mohammed V University, Rabat, Morocco; Cardiovascular Department B, Ibn Sina Hospital, Mohammed V University, Rabat, Morocco; Cardiovascular Department B, Ibn Sina Hospital, Mohammed V University, Rabat, Morocco; Cardiovascular Department B, Ibn Sina Hospital, Mohammed V University, Rabat, Morocco; Cardiovascular Department B, Ibn Sina Hospital, Mohammed V University, Rabat, Morocco

## Abstract

Patients with rheumatic mitral stenosis (MS) often present complications such as atrial fibrillation and thrombus formation with significant morbi-mortality. Rarely, a free-floating ‘ball thrombus’ is found with possible catastrophic outcomes. We describe three cases of documented left atrial ‘ping-pong’ shaped ‘thrombus ball’ within MS: a 51 year old presented with acute heart failure with a fatal outcome due to the huge round thrombus closing the tight mitral valve, a 67-year-old and a 68-year-old male who were both urgently rushed to the operating room after accidental finding. The surgery was successful and consisted on mitral valve repair and thrombectomy. Our aim is to show that gigantic unattached thrombus ball within neglected rheumatism MS is a rare life-threatening entity, thus highlighting the importance of early diagnosis of such conditions present in endemic countries. A prompt surgery should be considered to avoid an eventual embolization and sudden death.

## INTRODUCTION

Rheumatic mitral valve diseases are a major health concern worldwide, especially in endemic countries. Its physio-pathogenesis follows a natural history stamped with multiple complications, especially when neglected, with substantial Morbi-mortality related to thromboembolic events. One common complication is blood clot formation in the left atrium (LA), with a significant incidence of 33% [1].

Several types depending on the shape and the mobility have been described, but thrombus ball is an entity that remains very rare, with potential disastrous outcomes. Eventually, it can be the cause of sudden death in the setting of tight mitral stenosis (MS).

We present three different cases of patients with an unknown MS, whose echocardiographic findings revealed huge mobile round masses with ‘bumper-like’ effect evoking thrombus.

### Case 1

**Figure 1:**
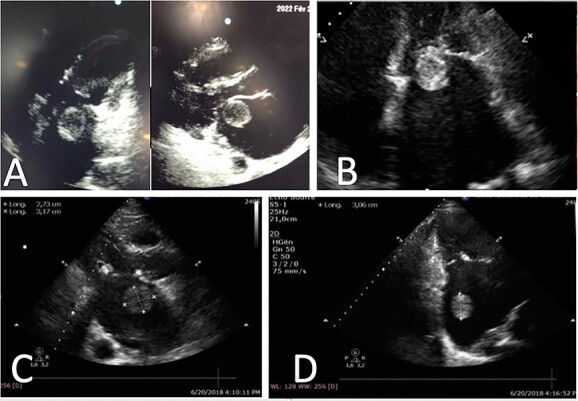
Representative echocardiography images of thrombus ball, (**A**) TTE views of a thrombus ball blocking the mitral valve (case 1), (**B**) Apical four chamber view on TTE showing a huge left atrial thrombus ball in contact with mitral valve (case 2), (**C**) and (**D**) Left atrial round thrombus on TTE (case 3).

We report the case of a 51-year-old Moroccan female, with history of untreated angina in her early childhood, who presented to the emergency room for stage IV dyspnea and palpitations. On admission, she was diagnosed with acute heart failure and rapid atrial fibrillation (AF) (180 bpm). The echocardiography revealed a very tight MS (mitral valve area: MVA: 0.7 cm^2^), as well as a dilated LA, with a huge floating thrombus (3 × 3 cm) spinning in the LA causing the intermittent obstruction of the valve (Fig. 1). The patient was urgently prepared for surgery, but unfortunately passed away from cardiac arrest, due to the thrombus closing the valve.

### Case 2

**Figure 2:**
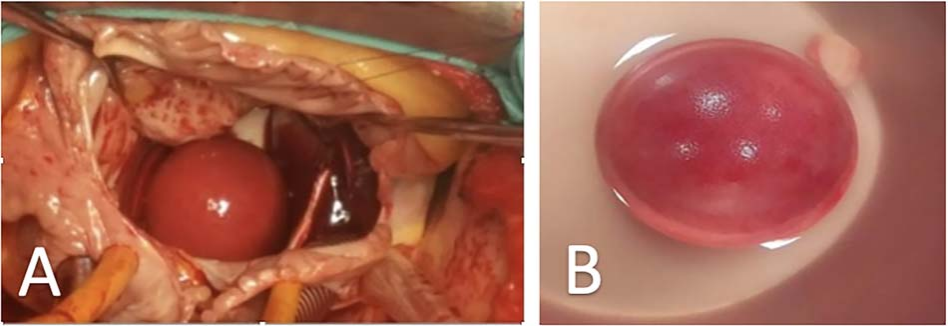
Representative images of surgical views of ‘thrombus ball’, (**A**) Surgical view of a smooth round ‘thrombus ball’ (case 2), (**B**) Thrombus ball after surgical removal (case 3).

This is the case of a 67-year-old male, with an unknown rheumatic MS. The patient was presented to our unit after aggravating dyspnea along with rapid AF. A transthoracic echocardiography (TTE) was performed, and showed tight MS with commissural fusion (MVA at 0.9 cm^2^ by planimetry), and a huge ‘ping-pong’ shaped mobile thrombus in the LA (Fig. 1; video 1). No regurgitation was objected. Surgery was indicated and the patient benefited from mitral valve replacement in addition to thrombectomy with success (Fig. 2).

### Case 3

The third patient is a 68-year-old male, who consulted for dyspnea on exertion and palpitations. His physical examination found a mitral diastolic bearing as well as tricuspid regurgitation murmur. The TTE revealed a severe rheumatic MS (MVA: 0.6 cm^2^) with an important mobile thrombus in the LA (Fig. 1). An important tricuspid regurgitation was also discovered. Emergency cardiovascular surgery was performed, and the patient benefited from mitral valve replacement, tricuspid valvuloplasty and thrombectomy (Fig. 2). The post-operative period was uneventful.

## DISCUSSION

Left atrial thrombus within MS is a common complication. The dilatation of the LA is favorable to blood stasis that progresses slowly into a sludge and eventually a thrombus, with notable risk of systemic embolization. Other factors can precipitate clot formation such as AF, LA size, advanced age and the severity of MS [2]. In case of AF, the shape, size, number, location and mobility are very well correlated to embolic complications [3]. However, AF is not necessary as thrombus formation was also described in patients with sinus rhythm [4]. According to Leug *et al.*, thrombus with dimensions greater or equal to 1.5 cm or a movable thrombus in the LA were predictors of subsequent thromboembolism, making ‘thrombus ball’ a risky shape [5].

Rarely, the thrombus can be mobile and take a round or spherical form growing into a large ‘ping-pong’ free-floating ball. It is suggested that the mobility in a narrow space helps forming this shape, molding it into a rounded smooth mass. Another characteristic is the massive size attained by those masses, growing from small ‘ping-pong’ ball to billiard ball [6], obtained when irregularly shaped bits of detached mural thrombi continue to grow by successive accretions assuming their spherical shape.

The differential diagnosis could be made with unattached myxoma or neoplastic lesions, but the context and the histopathological exam were clear to confirm the thrombus nature.

Thrombus ball was first described by Wooden after conducting an autopsy on a 15-year-old female patient with MS. Since then, different modalities of imaging, from TTE to Magnetic Resonance Imaging have helped describing this unusual thrombus. However, TTE is still a key examination in diagnosing those enormous thrombi for its accessibility.

Systemic embolization can occur when the large thrombus happens to be shattered by the effect of multiple collisions with the atrial wall, making it possible for small pieces to cross the narrow valve. Surprisingly, the theory of embolization within free thrombus is poorly supported in the literature. In fact, the supposed endothelization of the surface should prevent an eventual fragmentation.

Also, when a thrombus ball is so large, it cannot cross the mitral valve into the general circulation. However, it is not less dangerous than other types of thrombi, as it can cause syncope, pulmonary congestion or even sudden death with a ball-valve closing effect. This was the unfortunate case of our first patient. Therefore, the importance of early diagnosis in patients with history of rheumatic diseases or untreated angina.

Surgical thrombectomy must be performed urgently to prevent complications, with a survival rate of 90%. Mitral valve replacement is indicated to treat valvular stenosis [7]. The second and third patient have been successfully operated with no complications.

Acute management of LA thrombus ball seems not to include anticoagulation or thrombolytic therapy, due to the risk of superficial fragmentation and embolization [8]. Nevertheless, some rare cases of disappearing floating thrombus with anticoagulants have been described. It can be a life-saving alternative when classic surgical approach is deemed infeasible, making percutaneous approach a viable option after total disappearance of the thrombus. Anticoagulation before cloth formation at the stage of spontaneous contrast in case of MS with very dilated LA should be discussed, but there is no clear recommendation about it. Surgery, however, should be as urgent as possible.

## CONCLUSION

Thrombus ball is a rare and life-threatening entity complicating rheumatic MS. While complications arising from their unstable nature such as acute systemic embolism are mostly feared, thrombus ball can be particularly dangerous for causing syncope and sudden death. Urgent surgical management is recommended.

## Supplementary Material

video_rjad266
